# Rethinking stress in education: cortisol and DHEA-S biomarker outcomes of a mindset intervention

**DOI:** 10.3389/fnbeh.2025.1685319

**Published:** 2025-12-09

**Authors:** Ari Langrafe Junior, Luiz Claudio Fernandes, Evandro Moraes Peixoto, Anita Nishiyama

**Affiliations:** 1 Department of Physiology, Federal University of Paraná, Curitiba, Brazil; 2 Department of Psychology, São Francisco University, São Paulo, Brazil

**Keywords:** stress mindset, cortisol, DHEA-S, teachers, stress intervention, Stroop test, cognitive performance, psychoneuroendocrinology

## Abstract

**Objective:**

Teacher stress is a global concern with significant consequences for health, performance, and educational quality. While most studies address stress as a harmful phenomenon, emerging evidence suggests that an individual’s mindset toward stress can influence both psychological and physiological outcomes. This study investigated whether a brief video-based mindset intervention could alter stress perception and modulate biological stress markers among public school teachers in Brazil.

**Methods:**

A randomized controlled trial was conducted with 63 teachers allocated into intervention (*n* = 32) and control (*n* = 31) groups. The intervention group received an 8-days series of short educational videos developed by Stanford University’s Mind and Body Lab, designed to promote a growth-oriented stress mindset. Measures included the Stress Mindset Measure (SMM), Perceived Stress Scale (PSS-10), Stroop Color Task, and salivary biomarkers (cortisol and DHEA-S). Data were collected at baseline, post-intervention, and 30-days follow-up. Analyses included repeated measures ANOVA and *t*-tests.

**Results:**

The intervention significantly improved stress mindset scores immediately after the intervention and at follow-up (*p* < 0.001; ε^2^ = 0.664), with no change in the control group. Cortisol concentrations decreased significantly in the intervention group post-intervention (*p* = 0.004; ε^2^ = 0.262), though the effect was not maintained after 30 days. No significant changes were observed in DHEA-S levels. Additionally, cognitive performance on the Stroop incongruent task improved significantly in the intervention group (*p* = 0.003; *d* = 0.565), suggesting enhanced executive functioning under stress.

**Discussion:**

The findings support the effectiveness of a brief, low-cost intervention in shifting stress mindsets and producing acute physiological and cognitive benefits. However, the transient nature of the hormonal response underscores the need for sustained or complementary strategies to reinforce long-term stress resilience. This study highlights the value of mindset-based approaches in educational settings and their potential for improving teacher well-being through psychoneuroendocrinological mechanisms.

## 1 Introduction

According to the World Health Organization (2022), mental health is estimated to be one of the leading causes of illness among workers worldwide, resulting in a cost in lives that fail to promote the well-being of society, especially in basic education, which should be a healthy and supportive environment. Poor stress management in the workplace can lead to serious health problems. Education, especially in the school environment, is one of the most affected areas, with worrying teacher absenteeism that affects not only teachers but the entire school community (Ribeiro Gomes and Freitas, 2023).

In recent years, especially in the post-pandemic period, education has experimented with new strategies through new models of technology-mediated education. This has affected students, but especially teachers, who have had to adapt technically and technologically while dealing with the pressure to maintain educational standards. As a result, teachers reported high levels of stress, as observed in the study by Ozamiz-Etxebarria et al. (2023), conducted with primary school teachers in the Basque Country, Spain, during the post-pandemic period of COVID-19, which identified an overall prevalence of stress of 52% among participants. The study used standardized instruments to measure perceived stress and highlighted that factors such as work overload, emotional instability, and challenges in school demands were the main contributors to this situation.

Teachers constitute a particularly stressed professional group and are more affected than the average population by psychological complaints (Scheuch et al., 2015). Despite all the work-related stress, especially during the pandemic, many teachers managed to maintain a positive, optimistic, and proactive outlook, even in the face of barriers and difficulties arising from the changes brought about by social distancing and technology-mediated education (Grandisoli et al., 2023).

Thus, the question was asked: What is the dysfunctional factor that causes some teachers to become ill and others to thrive in stressful work environments? The answer may not lie in the amount of stress experienced, but in the way stress is experienced. The mindset toward stress argues that how we interpret stress, whether we see it as harmful or as a means for personal growth and development – can significantly influence our physiological and psychological responses to stress (Crum et al., 2013). The hypothalamic-pituitary-adrenal (HPA) axis plays a central role in neuroendocrine regulation of the stress response. When faced with stressful situations, the HPA axis is activated, resulting in the release of cortisol, a hormone crucial for the maintenance of homeostasis. In humans, cortisol has been used as a biomarker for stress condition and its concentration changes in acute and chronic situations. During activation of the HPA axis, adrenocorticotropic hormone (ACTH) induces a secretion of cortisol and dehydroepiandrosterone (DHEA) along with its sulphated form (DHEA-S) which both hormones are androgen precursors. They counterbalance the catabolic effects of cortisol (Young et al., 2002).

Studies on the impacts of stress tend to approach stress almost always negatively. So far, research on teacher stress has relied primarily on questionnaires suitable for measuring subjective experiences of stress, ignoring essential data on physiological reactions to stress in real time (Wettstein et al., 2020). In Brazil, only one study was found linking perceived stress to cortisol, using a sample of 20 male participants from the State Basic Public Education Network, with data collected at home and at school (Silva, 2017). The complex interaction between the nervous and endocrine systems in modulating the stress response may show how changes in stress perception can directly influence hormone secretion and, consequently, the physical and mental well-being of teachers.

Few studies address a multi-methodological assessment, which uses assessment methods, including self-reports, observational measures, and physiological measures, to survey participants in their workplaces (Trull and Ebner-Priemer, 2013). Despite these advantages, multi-methodological assessments have been underutilized around the world, especially in Brazil.

This study sought to verify, through an intervention of short videos provided by the laboratory Mind and Body from Stanford University, whether the mindset toward stress in teachers is correlated with major job stressors, performance, hormonal response, and the science in terms of investigating stress physiology. To this end, a multi-methodological assessment was chosen, using psychological measurements, recording stress reactions, and collecting hormone samples from teachers in their own school environment, which allows for a much deeper understanding of stress, as teachers tend to react to stress differently when out of school (Wettstein et al., 2020).

The study sought to discover whether changes in mindset regarding stress can be perceived not only subjectively, through self-reports and questionnaires, but also objectively, through biomarkers and scales. At the same time, rapid interventions may seem more timely, as they require less time and effort. Thus, this study also investigated whether the effects of a 1-week intervention have lasting effects 30 days later, in terms of subjective aspects and the body’s physiological response.

Based on this objective and on previous research in stress mindset theory and psychoneuroendocrinology, we formulated the following *a priori* hypotheses: (1) Participants in the intervention group would show a significant improvement in stress mindset scores (SMM) compared to controls. (2) They would also exhibit an acute reduction in salivary cortisol concentrations following the intervention. (3) Secondary outcomes would include improved performance on the Stroop Color Task (reaction time in the incongruent condition), decreased perceived stress scores (PSS), and possible variation in DHEA-S concentrations.

The primary outcomes were: (1) Change in stress mindset (SMM) scores; (2) Change in salivary cortisol concentrations. The secondary outcomes were: (1) Performance on the Stroop Task; (2) Perceived Stress Scale (PSS) scores and (3) DHEA-S concentrations.

The present trial extends prior stress-mindset work by embedding a brief, low-cost intervention within the authentic school context; coupling mindset change with executive control under conflict (Stroop) and salivary biomarkers collected at standardized times; and targeting teacher-specific stressors–emotional labor, crowding, discipline and voice demands, policy/assessment pressures–that are not well represented in general adult samples. This multi-method, randomized, naturalistic design in a middle-income setting advances the translational bridge from laboratory paradigms to educational practice.

## 2 Materials and methods

### 2.1 Ethics statement

To conduct this study, permission was obtained from the Paraná State Department of Education and approval was granted by the Research Ethics Council CEP/SD – UNICESUMAR (Approval No. 5.711.930).

### 2.2 Participants

Teachers from public elementary schools in the metropolitan region of Curitiba in South Brazil, were invited to participate in the study. Participants were selected through the communication channels of the Department of Education. As with the inclusion criteria, the teacher should be working in the classroom and not be away for any reason. The exclusion criterion was the expression of a desire not to participate in the study.

Participants were randomly allocated into two groups. The random allocation sequence was generated by a psychologist responsible for applying the protocol, who was not involved in the evaluation of results, tabulation, or data analysis.

We examined baseline characteristics stratified by group (sex, age, years of teaching experience, educational level, weekly workload, and baseline SMM and PSS-10) and tested between-group differences at baseline. No statistically significant differences were observed.

### 2.3 Groups intervention and control

The research design followed an experimental model of parallel groups, experimental and control, based on the proposal by Crum et al. (2013), which demonstrated that a change in mindset regarding stress can lead to improvements in physiological, emotional, and behavioral responses to stress. Additional studies corroborate this perspective, such as that by Jamieson et al. (2010) which showed that cognitive reappraisal of physiological arousal–interpreting stress signals as helpful rather than harmful–improves performance in stressful situations. Similarly, Jamieson et al. (2012) demonstrated that this approach is also effective in high-pressure contexts and decision-making under stress. Based on these findings and using G*Power software, the sample size was calculated considering a medium effect size (*d* = 0.66), as reported in the intervention studies on stress mindset conducted by Crum et al. (2013). The calculation indicated that at least 10 participants per group would be needed to achieve a statistical power of 0.95 for the analysis.

Stratified block randomization was used. The intervention group (*n* = 32) received the intervention, called “Rethink Stress,” consisting of short videos presented in sequence over eight consecutive days. The intervention consisted of a series of short videos provided by Stanford University’s Mind and Body Lab, translated and subtitled into Portuguese. These videos explore how the interpretation of stress can affect physical and mental health.

The other group watched videos on general health and was considered the control group (*n* = 31). To conduct the research, data were collected at a public high school in Paraná, where all participants were able to go at the end of the school day. Arrival was scheduled for 6:00 p.m., with questionnaires and tests beginning at 6:20 p.m. and saliva collection scheduled for 7:00 p.m. Participants were instructed to avoid coffee for 2 h before the sessions, as well as strenuous physical exercise, alcoholic beverages, and the use of non-prescription medications.

The teachers who participated in this study were affiliated with the Paraná State Education Network and were recruited beginning in March of the 2023 school year, with the intervention beginning 30 days later. Of the 72 participants who initially agreed to take part in the study, 63 were considered eligible, as they were working in the classroom at the time of data collection and had not taken leave for any reason. The participants were then randomized into two groups: the intervention group, with 32 teachers, and the control group, with 31 teachers. During the study, there were two dropouts in the intervention group, and all laboratory tests could be processed. In the control group, there were six dropouts, and two laboratory tests could not be analyzed. Thus, the final analysis included 30 teachers in the intervention group and 23 in the control group.

#### 2.3.1 Questionnaries

Initially, participants answered three short questionnaires: the Stress Mindset Measure (SMM), the Perceived Stress Scale (PSS-10), both validated in Brazil (Silva et al., 2022; Machado et al., 2014), and a biopsychosocial questionnaire, composed of a multidisciplinary approach that included biological, psychological, and social dimensions. The Stress Mindset Measure (SMM) was used to determine the degree of each stress factor, measuring the degree of emotions according to a scale and mapping categories of stress and predominant behaviors in the face of stress. At the same time, the Perceived Stress Scale (PSS-10) was used, a psychological tool developed to measure the degree of stress a person feels and how overwhelmed people feel about life’s demands compared to how much they feel they can cope with these demands.

#### 2.3.2 Simulating stress: Stroop Color Test

After answering the questionnaires, participants took the computerized Stroop Color Test, developed by the research team based on the classic model validated in the literature (Cohen et al., 1990). The test is widely used to assess selective attention, inhibitory control, and cognitive conflict processing, and consists of three tasks: word reading, color naming, and an interference condition. It has good internal consistency (α between 0.53 and 0.87) and proven validity in studies with clinical and normative populations (Fernandes, 2013). The version used in this study followed the parameters of the traditional test, adapted for computerized application, preserving its essential psychometric characteristics. The incongruent task can mimic real-life situations in which there is an overload of conflicting information or high cognitive demands, better reflecting the stressful conditions of everyday life. We explored this test in the context of our research to examine the body’s response to cognitive stress by measuring hormone secretion immediately after the test.

#### 2.3.3 Biomarkers

Saliva is a good source to test several substances, including hormones because is a non-invasive procedure and easily collected not requiring any needle. The measurement of cortisol and dehydroepiandrosterone sulfate (DHEA-S) in saliva was performed using the standard Salivette^®^ device (Sarstedt AG & Co., Germany). Participants were instructed to avoid food, beverages (except water), and toothbrushing for up to 2 h prior to collection to ensure sample integrity and prevent contamination such as gingival blood. Laboratory analyses were conducted in a certified laboratory. Salivary cortisol levels were determined by electrochemiluminescence using Roche Diagnostics^®^ kits (Germany), recognized for their high sensitivity and specificity in the analysis of steroids in the salivary matrix. In saliva, its levels reflect the biologically active fraction, not bound to proteins, and are sensitive to variations in the hypothalamic-pituitary-adrenal axis. The reference values for the collection time (between 4 p.m. and 8 p.m.) are below 0.252 μg/dL. Salivary DHEA-S was measured by chemiluminescence using Salimetrics^®^ kits (USA) specific for the salivary matrix, with adequate sensitivity for the detection of sulfated steroids. Laboratory reference values are based on serum parameters and adjusted for age and sex, ranging, for example, from 23 to 266 μg/dL for women aged 31–40 years, and 70–495 μg/dL for men aged 41–50 years.

The protocol was applied at three time points: baseline (pre-intervention), post-intervention (8 days after sending educational videos), and a 30-days follow-up. Samples were always collected at the same time of day, at 7 p.m., to control for circadian variations in the hormones analyzed. This evening schedule standardized diurnal phase across sessions and followed a controlled cognitive challenge, optimizing comparability for cortisol detection while acknowledging that slower-changing steroids such as DHEA-S may be less sensitive to single-timepoint evening sampling.

#### 2.3.4 Intervention: rethink stress

This study evaluated the effectiveness of the intervention on changing mindsets regarding stress, comparing it with a control group and correlating the results with work-related illnesses and biopsychosocial aspects. The primary outcome was whether the “Rethink Stress” intervention could change the teachers’ scores, which indicate a debilitating or growth mindset toward stress, and subsequently their perception of stress.

Secondary outcomes included checking for a possible correlation between a change in mindset regarding stress and the participants’ ability to control attention and process cognitive conflicts. To this end, the Stroop Color Test was administered, and the results were correlated with the Stress Mindset Scale (SMS). The test was conducted to verify whether a change in mindset toward stress could result in a significant improvement within a stressful scenario.

Finally, it was verified whether changes in mindset toward stress would influence cortisol and DHEA-S hormone levels. To this end, saliva samples were collected to measure cortisol and DHEA-S, which were correlated with the SMS results after the Stroop Color Test. Saliva samples were collected at the beginning of the study and at the end of the 8-days intervention, with a new collection performed 30 days after the end of the intervention to verify the duration of the effect.

### 2.4 Analyses

Statistical tests were performed using GraphPad Prism version 8.0 for Windows (GraphPad Software, USA). Data are reported as means ± SD. All data were evaluated for normality (Shapiro-Wilk test) and homoscedasticity (Bartlett test). The Student’s *t*-test was used to test assess significant differences in performance on specific cognitive tasks (such as the Stroop Color Test) between groups. One way ANOVA with repeated measures was used to compare differences over time between the intervention and control groups. The use of these methods allowed the identification of statistically significant changes in mindset in relation to stress and physiological response (cortisol levels), validating the effectiveness of the proposed intervention. For significant values (95% CI), effect sizes (ε^2^ - epsilon squared) were added, where values greater than 0.05 demonstrate an average effect and values greater than 0.1 indicate a large effect.

## 3 Results

Intervention and control groups did not differ at baseline in sex distribution, age, years of teaching experience, educational level, weekly workload, or baseline SMM and PSS-10. This supports the adequacy of randomization.

The final sample consisted of 30 women and 23 men, totaling 53 participants, of whom 84.9% presented a debilitating mentality in relation to stress, with a higher prevalence among women than men. A growth mindset was observed in only 15.1% of teachers. Participants were adults across early- to late-career stages, ranging in age from 20 to 67 years. A comparison was made between stress mentality and biopsychosocial data, and statistically significant associations were found between stress mentality and workload (*p* < 0.05, ε^2^ = 0.071), health problems related to absences (*p* < 0.001, ε^2^ = 0.290), use of psychotherapeutic drugs (*p* < 0.01, ε^2^ = 0.128) (Table 1). Stress mentality was also compared with self-reported stress perception (*p* < 0.05, ε^2^ = 0.164) (Table 2).

**TABLE 1:** Distribution of participants according to sociodemographic data and stress mentality.

Variable	Mindset toward stress				p^a^/ε^2^ ^b^
	Debilitating		Growth		
	*n*	%	*n*	%	
	45	84.9	8	15.1	
Gender identity					0.188
Female	26	49.2	4	7.5	
Male	19	35.8	4	7.5	
**Age range**					
18–30	6	11.5	3	5.8	0.199
30–39	11	21.5	1	1.9	
40–49	17	32.7	1	1.9	
50–59	6	11.5	2	3.8	
60–75	4	7.7	1	1.9	
**Education**					
Incomplete higher education	4	7.7	1	1.9	0.063
Complete higher education	5	9.6	3	5.8	
Postgraduate	35	67.3	4	7.7	
**Workload**					
20 classroom hours per week	5	9.6	4	7.7	0.050* ε^2^ = 0.071
40 classroom hours per week	39	75.0	4	7.7	
**Years teaching**					
1–9 years	14	26.9	3	5.8	0.226
10–19 years	13	25.0	2	3.8	
20–29 years	14	26.9	1	1.9	
30–39 years	3	5.8	2	3.8	
**Employment contract**					
Statutory	19	35.8	2	3.8	0.559
Temporary	26	49.1	6	11.3	
**Health problems**					
None with absences	22	41.5	8	15.5	<0.001* ε^2^ = 0.290
1–2 absences	20	37.7	0	0.0
3 or more absences	3	7.7	0	0.0
**Makes use of psychotropic medication**					
Yes	9	17.0	0	0.0	0.011* ε^2^ = 0.128
No	36	67.9	8	15.1
**Sleep conditions**					
Good	22	41.5	5	9.4	0.743
Reasonable	17	32.1	2	3.8	
Poor	6	11.3	1	1.9	

^a^Anova to a factor

^b^Effect size (ε^2^≈ 0.06, medium effect; ε^2^≈ 0.14, large effect). * *p* < 0.05.

**TABLE 2:** Participants’ self-reports on work stress and stress mindset.

Variable	Mindset toward stress				p^a^/ε^2^ ^b^
	Debilitating		Growth		
	*n*	%	*n*	%	
	45	84.9	8	15.1	
**How much stress are you experiencing?**					
None	4	7.5	2	3.8	0.255
A moderate amount	35	66.0	6	11.3	
An extreme amount	6	11.3	0	0.0	
**What is the main source of stress?**					
Work	35	66.0	5	9.4	0.050* ε^2^ = 0.164
Other factors	13	18.9	3	5.7	
**How stressful is your work?**					
Not at all stressful	1	1.9	1	1.9	0.354
Moderately stressful	29	54.7	7	13.2	
Extremely stressful	15	28.3	0	0.0	

^a^Anova to a factor

^b^Effect size (ε^2^≈ 0.06, medium effect; ε^2^≈ 0.14, large effect). * *p* < 0.05.

### 3.1 The mindset shift

**FIGURE 1:**
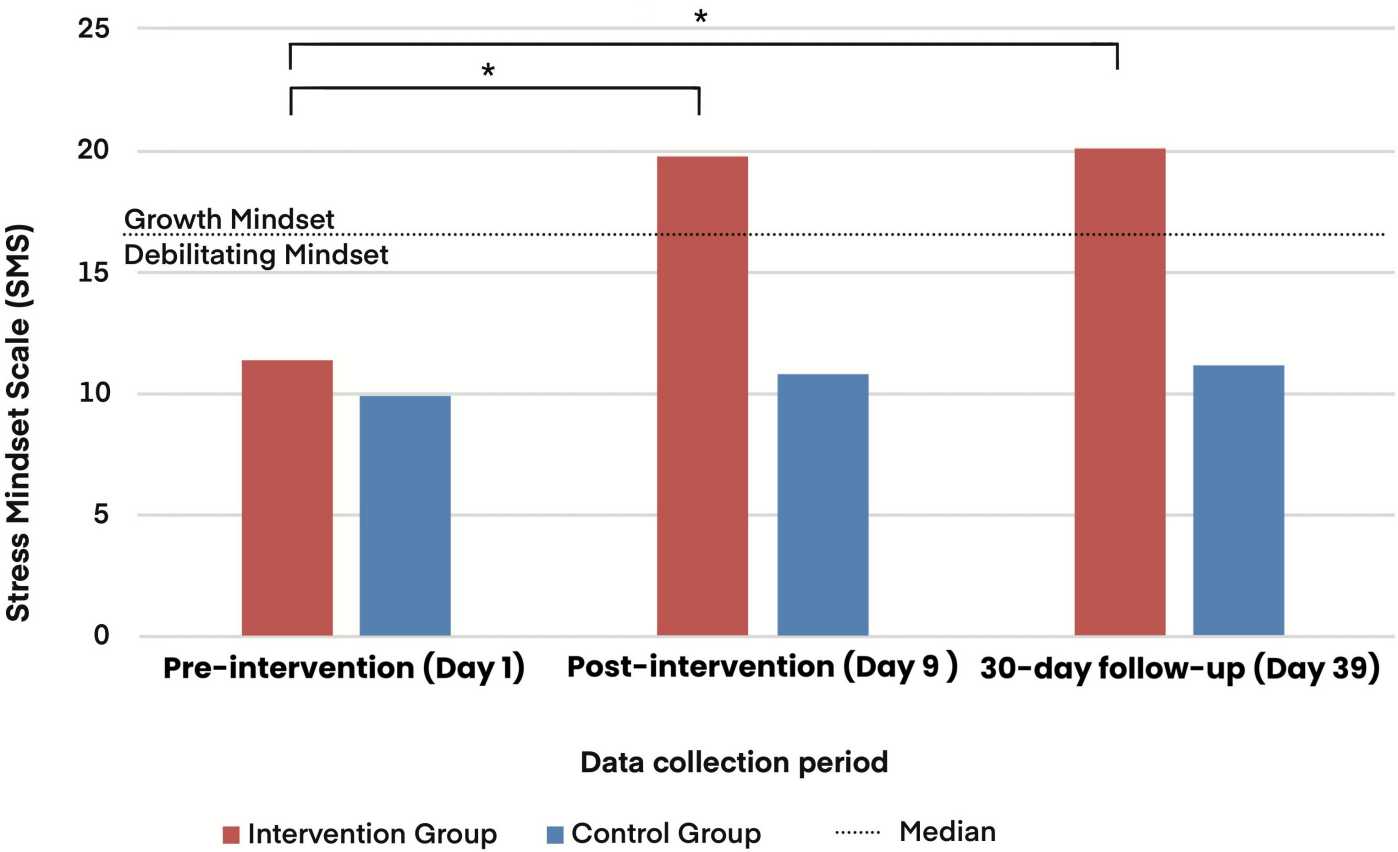
Mindset toward stress after the 30-days follow-up intervention in the intervention and control groups. The median of the scale divides the growth mindset from the debilitating mindset. * *p* < 0.05.

The results showed that the intervention group presented a significant change in stress mindset immediately after the intervention (*p* < 0.001; ε^2^ = 0.664), an effect that was maintained at the 30-days follow-up (*p* < 0.001; ε^2^ = 0.664), indicating a lasting effect of the intervention. In the control group, which watched neutral videos about general health, there was no significant change in stress mindset scores, either immediately after the intervention (*p* = 0.227) or at follow-up (*p* = 0.134) (Figure 1).

The change in mindset toward stress, therefore, may be helpful for educators, not only to manage their own responses to stress, but also to positively influence their approach to the challenges of teaching. Adopting this perspective requires, however, ongoing awareness and training about how personal beliefs about stress impact our bodies and minds, as well as strategies to reframe these beliefs in more positive and productive ways (Hecht et al., 2023; Greenberg et al., 2016).

### 3.2 Behavioral response to a stressor

To investigate the impact of the intervention on cognitive performance, a paired *t*-test was used to assess whether the change in mindset about stress affected response time on the Stroop Color Test. In the congruent test (with matching words and colors), there were no significant differences for either the intervention group (*p* = 0.062) or the control group (*p* = 0.066), indicating that performance on low-cognitive-demand tasks remained stable.

**FIGURE 2:**
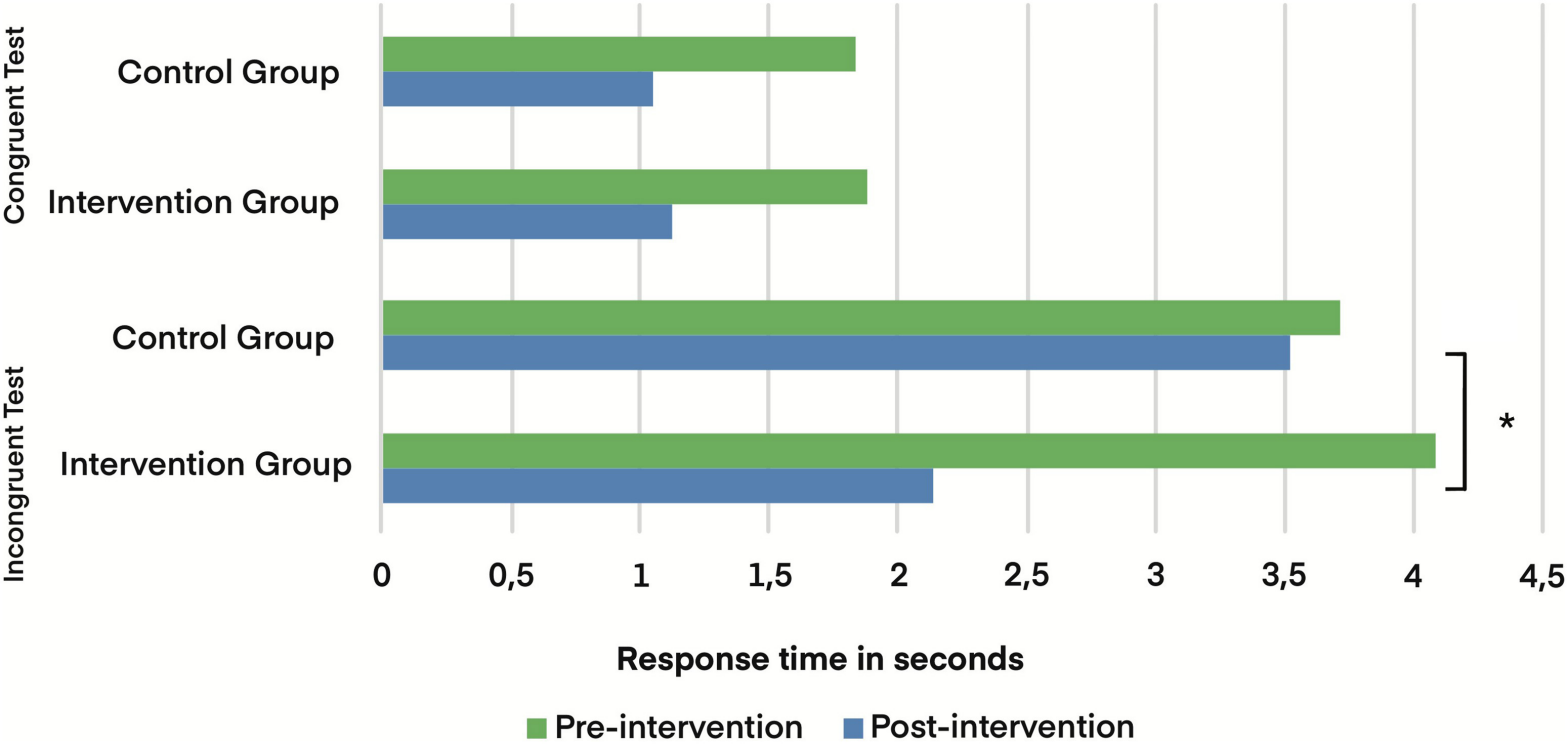
Response time in the congruent and incongruent test in the control and intervention groups. * *p* < 0.05.

On the other hand, in the incongruent test (with mismatched words and colors), the results indicated that the intervention group showed a significant improvement in performance, with a reduction in response time (*p* = 0.003; *d* = 0.565) that represents a moderate effect size, indicating that the intervention was not only statistically significant but also produced a relevant and consistent improvement in inhibitory control and selective attention, especially in tasks with higher cognitive demands. In the control group, no significant changes were observed (*p* = 0.456) (Figure 2). These results suggest that changing the mindset about stress not only modifies the perception of stress but also enhances performance in cognitive tasks that require greater conflict management and executive control.

### 3.3 Physiological response to a stressor

**FIGURE 3:**
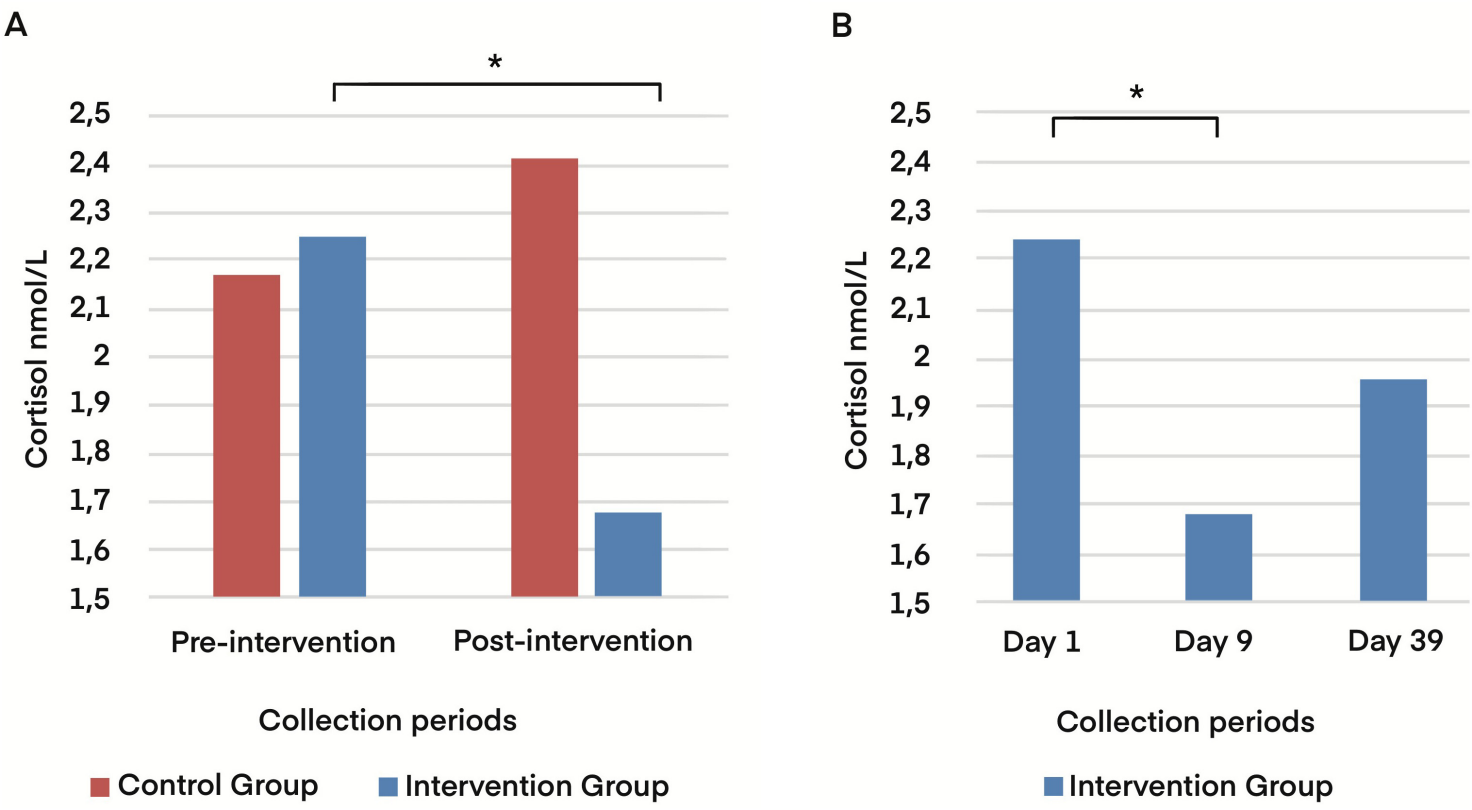
Neuroendocrine cortisol response after intervention in the control and intervention groups. (A) Salivary cortisol (nmol/L) at pre-intervention (Day 1) and post-intervention (Day 9) in the control and intervention groups. (B) Salivary cortisol (nmol/L) for the intervention group across pre-intervention (Day 1), post-intervention (Day 9), and 30-day follow-up (Day 39). * *p* < 0.05.

Regarding salivary cortisol, the paired Wilcoxon test, suitable for nonparametric data, was used to compare hormone concentrations before and after the intervention. In the control group, there was no significant change (*p* = 0.329), while in the intervention group, a significant reduction in cortisol concentrations was observed after the intervention (*p* = 0.004; ε^2^ = 0.262) (Figure 3A). DHEA-S concentration did not change between the groups (data not shown).

To assess the durability of the effect, a new measurement was performed in the intervention group 30 days after the end of the intervention (day 39). Our results indicated that the effect of intervention was lost and start climbing, with a non-significant difference (*p* = 0.178) (Figure 3B).

## 4 Discussion

### 4.1 Structural determinants of teacher stress

Beyond reproducing general stress-mindset effects, our contribution is teacher-specific. Teaching involves distinctive stress exposures–continuous emotional labor, simultaneous monitoring of large classes, voice load, frequent discipline decisions, multi-tasking under time constraints, and external policy/assessment demands. By situating a brief mindset intervention within this ecology and linking it to inhibitory control (Stroop incongruent) and HPA-axis indices, we show how appraisal shifts may translate into classroom-relevant cognitive performance and acute endocrine modulation in an occupational group with high burnout risk.

This study highlighted the feasibility of interventions to change the mentality regarding stress among teachers, considering that most participants had a debilitating mindset about stress, associated with factors such as long working hours, health problems, medication use, and the perception of stress as inherent to teaching activity. Beyond mindset-focused approaches, skills-based supports such as classroom management training can buffer early-career “reality shock” and improve teachers’ adjustment, complementing individual-level interventions (Dicke et al., 2015).

These findings are consistent with previous studies, such as those by Greenberg et al. (2016), which highlight that chronic stress in the school environment is strongly related to teacher illness, reduced professional effectiveness, and increased absences due to health reasons. Similarly, Agyapong et al. (2022) observed that teachers subjected to high levels of emotional demand and workload have higher rates of psychotropic medication use and burnout symptoms.

These convergences reinforce that teacher stress should not be understood only as an individual reaction, but as a manifestation of structural working conditions that impact both the health of professionals and the quality of education. In this context, interventions focused on changing the mindset about stress emerge as a promising, low-cost, and scalable strategy capable of mitigating the negative effects of stress, promoting greater well-being and resilience in the school environment.

From a neurobiological perspective, mindset-based interventions are believed to influence stress responses through cognitive reappraisal mechanisms involving top-down regulation of the hypothalamic-pituitary-adrenal (HPA) axis. Neuroimaging studies have shown that reinterpreting the meaning of a stressor can engage the prefrontal cortex (PFC)–particularly the dorsolateral and ventromedial regions–which exerts inhibitory control over the amygdala and modulates hypothalamic activity responsible for initiating cortisol secretion (Jamieson et al., 2012; Crum et al., 2013). This cortico-limbic pathway is central to emotional regulation and stress processing. When individuals adopt a more adaptive or “growth” mindset regarding stress, these cortical circuits may facilitate a reduced threat appraisal, lowering the reactivity of limbic regions and ultimately attenuating activation of the HPA axis. This theoretical model aligns with the observed acute decrease in cortisol concentrations post-intervention in the current study, suggesting that brief mindset training may enhance the brain’s capacity to modulate physiological responses to stressors through cognitive-emotional control.

### 4.2 Changing mindsets: a promising intervention

The intervention applied to the experimental group revealed a significant change in mindset regarding stress, an effect that lasted for 30 days. This finding corroborates the research by Agyapong et al. (2023), Chesak et al. (2019), Song et al. (2020), who demonstrated that interventions focused on modifying the perception of stress can result in lasting changes in mindset and, consequently, in well-being (Yeager et al., 2019).

### 4.3 Divergence between mindset and perception of stress

In this study, using the PSS-10 scale, it was found that teachers continued to recognize stress as part of their work, while the SMM indicated that teachers may experience stress differently after a change in their mindset toward stress. The thought that stress can have beneficial aspects is fundamental for the improvement in mentality toward stress and may be essential for the continued professional development of teachers (Schreiber and Schotanus-Dijkstra, 2024). The results indicate that although stress mindset has changed, teachers’ overall perception of stress has not changed significantly. This suggests that improving the mindset toward stress does not necessarily reduce the perception of stress, as studies in the field have found (Jamieson et al., 2020) but may change the way stress is experienced and managed.

Conceptually, SMM indexes beliefs about the meaning and potential utility of stress (meta-appraisal), whereas PSS-10 reflects perceived load and uncontrollability over recent weeks. A brief, content-specific intervention can shift meta-appraisals without immediately altering contextual demands; consequently, mindset improved while PSS-10 remained stable. Over longer horizons, SMM changes may facilitate adaptive coping and affect regulation that, in turn, reduce perceived stress. Longitudinal designs (≥ 3–6 months) using mixed-effects models should test whether SMM improvements prospectively mediate gains in coping/executive control and downstream reductions in PSS-10. Studies such as those by Jamieson et al. (2010) have explored how cognitive reappraisal can alter emotional and physiological responses to stress, indicating that changes in stress perception may require more prolonged or intensive interventions.

This study also showed that changing one’s mindset toward stress can improve performance in situations of cognitive conflict, simulated in the incongruent Stroop test, in which there is an overload of conflicting information or elevated cognitive demands. This finding is in line with the literature that suggests that the way stress is interpreted can directly influence performance, especially in tasks that require high cognition and emotional resilience (Yeager et al., 2019).

### 4.4 Neuroendocrine evidence of change: cortisol and DHEA-S

As suggested by Wettstein et al. (2020), cortisol and DHEA-S measurements were taken directly in the school environment to complement the multi-methodological approach. Salivary cortisol is widely recognized as a valuable physiological biomarker for measuring stress states due to its close relationship with the body’s response to stress. Salivary cortisol measurement has proven to be a non-invasive and practical window for assessing the activity of the hypothalamic-pituitary-adrenal (HPA) axis, which plays a central role in the stress response. Interindividual variability in cortisol and DHEA-S responses may also reflect sex-specific patterns of HPA activation reported in the literature (Kudielka and Kirschbaum, 2005).

Importantly, cortisol and DHEA-S differ in kinetics and functional roles: cortisol exhibits rapid, phasic reactivity to appraisal and challenge, whereas DHEA-S shows a longer half-life and more tonic, resilience-related properties. Our fixed evening sampling (19:00) controlled for diurnal phase and aligned with a standardized cognitive challenge, a design that favors comparability across sessions and detection of cortisol shifts but may under-detect slower-changing steroids such as DHEA-S. Future work should employ multi-sample diurnal profiles (including awakening and the cortisol awakening response), post-stressor time-courses (e.g., +15 and +30 min), and ratio metrics (cortisol: DHEA-S) to index anabolic–catabolic balance more comprehensively.

The HPA axis is activated in response to perceptions of stress, leading to the release of cortisol. This study demonstrated that improving the mindset toward stress led to a decrease in cortisol secretion in response to a stressful or high-performance situation, allowing for an adequate response to the daily challenges of teaching and preventing chronic stress.

The results of this study demonstrated that intervention based on changing the mindset about stress was effective in reducing salivary cortisol concentration, indicating an acute modulation of the physiological response to stress. This reduction, observed shortly after the intervention, is in line with evidence from literature showing that cognitive strategies for reinterpreting stress can directly impact the hypothalamic-pituitary-adrenal (HPA) axis, reducing cortisol secretion (Crum et al., 2013; Jamieson et al., 2020).

### 4.5 Short-term effects and the need for sustained strategies

However, the fact that cortisol concentrations did not remain reduced during the 30-days follow-up suggests that, although brief interventions are effective in generating immediate physiological changes, their effects may be transient, especially if there is no continuous reinforcement of the content and practices learned.

Although the observed reduction in cortisol levels immediately following the intervention is encouraging, the absence of a sustained effect at the 30-days follow-up suggests that brief interventions may require reinforcement strategies to maintain physiological gains. It is possible that a 1-week video-based intervention, while sufficient to influence mindset and short-term responses, may not provide the intensity or continuity needed to promote lasting neuroendocrine adaptation. To enhance sustainability, future programs should consider implementing booster sessions, integrating mindset training into ongoing school health policies, or combining cognitive reappraisal approaches with complementary strategies such as mindfulness-based stress reduction or cognitive-behavioral techniques. Studies have demonstrated that such integrative approaches can foster more durable changes in both psychological and physiological domains (Chesak et al., 2019; Song et al., 2020). These findings underscore the importance of not only initiating change but also sustaining it through supportive structures embedded in educational environments.

Previous studies corroborate this observation, indicating that longer interventions, continuous follow-up, or complementary strategies such as coping training, mindfulness, or psychosocial support are necessary for sustained regulation of the HPA axis (Sanetti et al., 2021).

This finding reinforces the importance of incorporating, in educational and organizational contexts, not only specific interventions, but also permanent structures to support mental health, which can promote the maintenance of physiological and psychological benefits over time.

The attenuation of the cortisol effect at 30 days suggests that mindset reframing, while sufficient to shift appraisal and immediate HPA responsivity, may require periodic reinforcement to consolidate into longer-term endocrine adaptation. Feasible school-based strategies include monthly 10–15-min booster videos, brief in-person micro-sessions, and integration with skills-based modules (e.g., mindfulness, cognitive reappraisal drills, or CBT-informed coping). A pragmatic stepped-care design–brief mindset module plus low-dose boosters with optional skills add-ons–could maintain cognitive-emotional gains while supporting durable physiological regulation and should be tested in future trials.

The study by Scherzinger et al. (2018), Janssen et al. (2018) collected eight saliva samples from teachers throughout the day to determine cortisol and alpha-amylase secretion with perceived stress assessments and recorded anger ratings and found significantly higher morning cortisol concentrations on workdays compared to days off. In this study, nighttime cortisol was measured at baseline to detect changes in secretion rates after a day’s work.

The results indicated that the intervention positively influenced cortisol concentration but not those of DHEA-S. While the intervention yielded a significant reduction in salivary cortisol levels, no significant changes were observed in DHEA-S concentrations. This null finding warrants deeper consideration. DHEA-S is often considered a marker of long-term resilience and anabolic balance, and it may not respond as readily to acute stress modulation as cortisol does (Izawa et al., 2008; Morgan et al., 2004). Unlike cortisol, which fluctuates rapidly in response to stressors, DHEA-S has a longer half-life and more stable secretion patterns, potentially requiring longer interventions to produce measurable changes (Kroeper et al., 2022). Moreover, although its circadian rhythm is less pronounced than that of cortisol, it still contributes to interindividual variability in short-term studies (Scherzinger et al., 2018). Previous evidence suggests that DHEA-S may play a greater role in stress adaptation under chronic conditions or sustained behavioral change (Maninger et al., 2008; Morgan et al., 2004). Thus, future research should explore whether extended interventions or multimodal approaches (e.g., combining mindset shifts with mindfulness or lifestyle changes) might induce shifts in this biomarker.

Dehydroepiandrosterone-S has been associated with stress resilience and more effective recovery after exposure to stress. This finding reinforces previous studies that point to the complexity of hormonal responses to stress and the need to better understand the underlying mechanisms that regulate these responses (Maninger et al., 2008).

### 4.6 Implications for policy and future research

For the intervention to have a lasting effect on mental well-being, it may be necessary to incorporate a change in mindset regarding stress in everyday life. Future research should investigate quick interventions versus intensive interventions with longer follow-up times to examine whether and how stress mindset can be changed in a sustainable way and promote mental health in the general population (Kroeper et al., 2022).

### 4.7 Limitations of the study

Despite its contributions, this study has some limitations that should be considered. The relatively small sample size may limit the generalization of the results to other populations. In addition, data collection was conducted in a specific geographical and institutional context (basic education teachers in Paraná), which may restrict the applicability of the findings to different educational settings. Another point is that, although the intervention generated immediate and sustained effects on mindset and cognitive performance, the effect on cortisol physiological regulation was not maintained in the follow-up, indicating that brief interventions may require complementary strategies or periodic reinforcement to maintain biological effects.

Although the study demonstrates promising effects, the modest sample and single-region, single-system recruitment constrain external validity. Volunteer bias and regional work-culture features may limit generalization. Future multi-site replications with stratified sampling across regions, school types, and teacher career stages are needed to validate robustness and transportability of effects.

Finally, the use of self-report instruments may be subject to social desirability bias and subjective perception, despite being complemented by objective performance measures and biomarkers.

## 5 Conclusion

This study contributes to the literature on stress management among teachers by highlighting the effectiveness of interventions based on changing mindsets about stress. Through a multi-methodological approach, we were able to demonstrate that this change has an impact not only on subjective perception but also on objective responses, such as reduced cortisol secretion, highlighting the benefits of a more adaptive response to stress and, consequently, preventing the development of chronic stress.

We have proven that an improved perspective on stress is not only possible but also sustainable, promoting significant improvements in teachers’ well-being, cognitive performance, and physiological response. The results suggest that by adopting a more positive and functional view of stress, individuals can not only mitigate its negative effects but also strengthen resilience, optimism, and a sense of self-efficacy in the face of challenges. Rethinking stress, therefore, emerges as a viable strategy for promoting a healthier, more productive, and more engaged life, recognizing that our response to stress is largely a matter of perspective and interpretation, rather than fixed or unchangeable circumstances.

When educators begin to view the challenges of teaching as opportunities for personal and professional growth, they not only transform their own experience, but also become models of emotional regulation and resilience for their students, teaching them, explicitly or implicitly, that stress can be a catalyst for learning, development, and overcoming obstacles.

## Data Availability

The raw data supporting the conclusions of this article will be made available by the authors, without undue reservation.
